# SWI/SNF‐deficiency defines highly aggressive undifferentiated endometrial carcinoma

**DOI:** 10.1002/cjp2.188

**Published:** 2020-10-30

**Authors:** Basile Tessier‐Cloutier, Mackenzie Coatham, Mark Carey, Gregg S Nelson, Sarah Hamilton, Amy Lum, Robert A Soslow, Colin JR Stewart, Lynne M Postovit, Martin Köbel, Cheng‐Han Lee

**Affiliations:** ^1^ Department of Pathology and Laboratory Medicine University of British Columbia Vancouver Canada; ^2^ Department of Oncology University of Alberta Edmonton Canada; ^3^ Department of Obstetrics and Gynecology University of British Columbia Vancouver Canada; ^4^ Department of Gynecologic Oncology Tom Baker Cancer Centre and University of Calgary Calgary Canada; ^5^ Department of Radiation Oncology BC Cancer Vancouver Canada; ^6^ Department of Pathology Memorial Sloan Kettering Cancer Center New York NY USA; ^7^ Department of Histopathology, King Edward Memorial Hospital and School for Women's and Infants' Health University of Western Australia Perth Australia; ^8^ Department of Pathology and Laboratory Medicine Calgary Laboratory Services and University of Calgary Calgary Canada; ^9^ Department of Laboratory Medicine and Pathology Royal Alexandra Hospital and University of Alberta Edmonton Canada; ^10^ Department of Pathology and Laboratory Medicine BC Cancer Vancouver Canada

**Keywords:** dedifferentiation, undifferentiated endometrial cancer, SMARCA4, SMARCB, ARID1A, ARID1B

## Abstract

Dedifferentiated/undifferentiated endometrial carcinoma (DDEC/UEC) is an endometrial cancer characterized by the presence of histologically undifferentiated carcinoma. Genomic inactivation of core switch/sucrose nonfermentable (SWI/SNF) complex proteins was recently identified in approximately two‐thirds of DDEC/UEC. The aim of this study was to delineate the clinical behavior of SWI/SNF‐deficient DDEC/UEC in comparison to SWI/SNF‐intact DDEC/UEC. The study cohort consisted of 56 SWI/SNF‐deficient DDEC/UEC (2 *POLE*‐mutated), which showed either SMARCA4 (BRG1) loss, ARID1A/1B co‐loss, or SMARCB1 (INI1) loss in the undifferentiated tumor, and 26 SWI/SNF‐intact DDEC/UEC (4 *POLE*‐mutated). The average age at diagnosis was 61 years for patients with SWI/SNF‐deficient tumors and 64 years for SWI/SNF‐intact tumors. Mismatch repair (MMR) protein deficiency was seen in 66% of SWI/SNF‐deficient and 50% of SWI/SNF‐intact tumors. At initial presentation, 55% of patients with SWI/SNF‐deficient tumors had extrauterine disease spread in contrast to 38% of patients with SWI/SNF‐intact tumors. The 2‐year disease specific survival (DSS) for stages I and II disease was 65% for SWI/SNF deficient tumors relative to 100% for SWI/SNF‐intact tumors (*p* = 0.042). For patients with stages III and IV disease, the median survival was 4 months for SWI/SNF‐deficient tumors compared to 36 months for SWI/SNF‐intact tumors (*p* = 0.0003). All six patients with *POLE*‐mutated tumors, including one with stage IV SWI/SNF‐deficient tumor were alive with no evidence of disease. Among the patients with advanced stage SWI/SNF‐deficient tumors, 68% (21 of 31) received adjuvant or neoadjuvant chemotherapy (platinum/taxane‐based) and all except the patient with a *POLE*‐mutated tumor (20 of 21) experienced disease progression either during chemotherapy or within 4 months after its completion. These findings show that core SWI/SNF‐deficiency defines a highly aggressive group of undifferentiated cancer characterized by rapid disease progression that is refractory to conventional platinum/taxane‐based chemotherapy. This underscores the importance of accurate clinical recognition of this aggressive tumor and the need to consider alternative systemic therapy for these tumors.

## Introduction

Dedifferentiated/undifferentiated endometrial carcinoma (DDEC/UEC) is defined in the 2020 World Health Organization (WHO) classification as a malignant epithelial neoplasm with no overt cell lineage differentiation [1]. In the case of dedifferentiated carcinoma, the undifferentiated carcinoma occurs in combination with a clonally related typically International Federation of Gynecology and Obstetrics (FIGO) grade 1 or 2 endometrioid‐type carcinoma [2, 3], though the association with high‐grade endometrioid‐type carcinoma also occurs, albeit less commonly [4]. Studies that employed centralized pathology review using strict histologic criteria have demonstrated an aggressive clinical behavior for DDEC/UEC, with significantly worse outcome than International Federation of Gynecology and Obstetrics (FIGO) grade 3 endometrioid carcinoma [2, 5, 6, 7]. There are however inherent practical diagnostic challenges in separating FIGO grade 3 endometrioid carcinoma from an undifferentiated carcinoma histologically.

Genetically, DDEC/UEC frequently arises in a microsatellite unstable (MSI‐H)/mismatch repair (MMR) protein deficient setting [8, 9, 10]. In a series of recent studies, we and others identified frequent genomic inactivation of core components of SWI/SNF chromatin‐remodeling complex which resulted in the loss of expression of the corresponding proteins in about two‐thirds of DDEC/UEC [2, 10, 11, 12, 13, 14]. In the case of DDEC, the inactivating mutation and consequent protein loss was only observed in the undifferentiated carcinoma and not in the corresponding clonally related differentiated endometrioid carcinoma component [2, 3]. Loss of activity of these core SWI/SNF complex proteins is expected to abrogate function of the SWI/SNF complex, resulting in dysregulated chromatin remodeling and transcriptional control. While additional functional studies are needed to confirm a causal relationship between core SWI/SNF protein inactivation and the development of DDEC/UEC, there are several examples linking core SWI/SNF protein inactivation and the development of histologically and immunophenotypically undifferentiated malignancy. These include SMARCB1 (protein also known as INI1) inactivation in malignant rhabdoid tumor, atypical rhabdoid/teratoid tumor, epithelioid sarcoma and undifferentiated sinonasal carcinoma, and SMARCA4 (protein also known as BRG1) inactivation in small cell carcinoma hypercalcemic‐type of the ovary, SMARCA4‐deficient uterine sarcoma/malignant rhabdoid tumor of the uterus, rhabdoid undifferentiated lung carcinoma and gastrointestinal tract carcinoma [15, 16, 17, 18, 19, 20, 21, 22, 23, 24, 25, 26].

Clinically, we observed in our initial studies a trend for SWI/SNF‐deficient DDEC/UEC to be associated with worse disease‐specific survival (DSS) compared to SWI/SNF‐intact DDEC/UEC [2, 11, 12]. This suggests potentially important biologic differences between these tumors. Moreover, there has been growing development in drugs targeting chromatin remodeling, which has demonstrated efficacy in targeting tumors harboring SWI/SNF deficiency [27, 28, 29]. The current study aims to gain a better understanding of the clinical behavior of SWI/SNF‐deficient DDEC/UEC in comparison to morphologically defined DDEC/UEC without detectable SWI/SNF alterations herein designated as SWI/SNF‐intact DDEC/UEC.

## Materials and methods

### Study cohorts

The retrospective study included 82 cases of DDEC/UEC (64 of the 82 cases were previously described by our group) [11, 12]. These cases were identified through institutional pathology database search at Vancouver General Hospital (Vancouver, Canada), Royal Alexandra Hospital (Edmonton, Canada), Calgary Laboratory Services (Calgary, Canada), Memorial Sloan Kettering Cancer Centre (New York, NY, USA) and King Edward Memorial Hospital (Perth, Australia). All of the endometrial carcinomas included in this study were centrally reviewed (MK and CHL), fulfilling the morphologic features described by Silva *et al* [5, 6] Clinical information including demographics, staging (FIGO; International Federation of Gynecology and Obstetrics), treatment and disease course was obtained through review of medical records. The study was approved by the Institutional Review Boards (University of Alberta, Pro00042667).

### Immunohistochemistry

The expression of SMARCA4 (BRG1), SMARCB1 (INI1), ARID1A, ARID1B, and MMR proteins – MLH1, MSH2. MSH6, and PMS2 were examined in 64 reported cases by immunohistochemistry [2, 11]. The same immunohistochemical analysis was performed on the additional 18 cases. Immunohistochemistry was performed on whole tissue sections. The slides were incubated with antibodies to ARID1A (1:200, HPA005456, Sigma, Oakville, Canada), ARID1B (1:100, clone 2D2, H00057492‐M01, Abnova, Taipei, Taiwan), SMARCA4 (1:25, clone EPNCIR111A, ab110641, Abcam, Toronto, Canada), and SMARCB1 (1:50, 25/BAF47, 612 110, BD Biosciences, Mississauga, Canada); SMARCA4 and SMARCB1 were processed using Ventana Benchmark XT (Ventana Medical Systems, Tucson, AZ, USA), while ARID1A and ARID1B were processed using Dako Omnis Autostainer (Dako Canada ULC, Mississauga, Canada). The detection system used was the Bond polymer refine (Leica Microsystems, Wetzlar, Germany). For MMR proteins, the slides were incubated with MLH1 (DAKO clone ES05 1:100, Mississauga, Canada), MSH2 (NCL clone 25D12 prediluted, Concord, ON, Canada), MSH6 (BD Bioscience 44/MSH6 1:2000, Mississauga, Canada), PMS2 (BD Bioscience A16‐4 1:100, Mississauga, Canada), and processed using the Leica Bond Max platform (Leica Microsystems, Wetzlar, Germany) as per manufacturer's protocol with proprietary reagents. For interpretation, only nuclear protein expression in the undifferentiated carcinoma was assessed and the tumor was scored as showing intact expression if any tumor cell nuclei showed nuclear staining and deficient if the tumor nuclei were unstained in the presence of internal positive control immunoreactivity.

### POLE exonuclease domain mutation analysis

DNA extraction and analysis from formalin fixed paraffin embedded (FFPE) tumor samples were performed as previously described [30]. DNA purity and yield were determined using a NanoDrop 2000c spectrophotometer (Thermo Fisher Scientific, Waltham, MA, USA). Primer sets that cover the exonuclease domain of *POLE* in which mutations were previously identified in endometrial carcinomas were used to amplify exonuclease domain genomic regions – exon 9 (forward: 5′‐TGTTCAGGGAGGCCTAA TGG‐3′; reverse: 5′‐AACAAATACTAACAGTGGGG‐3′), exon 10 (forward: 5′‐GCTG CAATTCTGATCTGACG‐3′; reverse: 5′‐CAGCCTCTGACTTGTGCTGA‐3′), exon 11 (forward: 5′‐CTTCTGAACTTTGGGAGAGG‐3′; reverse: 5′‐CACCTCCTAAGTCG ACATGG‐3′), exon 12 (forward: 5′‐GCATTAGAGCCTGACCTGC‐3′; reverse: 5′‐ACAGCACAGTCTGCAAGAGG‐3′), exon 13 (forward: 5′‐CGGGATGTGGCTTAC GTGC‐3′; reverse: 5′‐TTGCATCTGTCTGTGTGGTG‐3′), exon 14 (forward: 5′‐TCTGTGCTTCACACTTGACC‐3′; reverse: 5′‐GACATCCACCTCCATTCAGC‐3′). PCR amplifications were performed as previously described using 50 ng genomic DNA and the primer sets using High‐Fidelity Tag DNA polymerase (Invitrogen, Carlsbad, CA, USA). PCR amplicons were then purified using Axygen™ AxyPrep Mag™ PCR Clean‐up Kits according to manufacturer's protocol (Axygen Biosciences, Union City, CA, USA) and resuspended in 30ul double‐distilled water. Direct bi‐directional sequencing was performed on a 3730xl DNA Analyzer (Applied Biosystems, Carlsbad, CA, USA) with 96 capillaries.

### Statistical analysis

Univariate disease‐specific survival analysis was performed by generating Kaplan–Meier curves and log‐rank statistics were applied (WinStat). A *P*‐value <0.05 was considered statistically significant.

## Results

### Clinical and pathologic features

**Figure 1:**
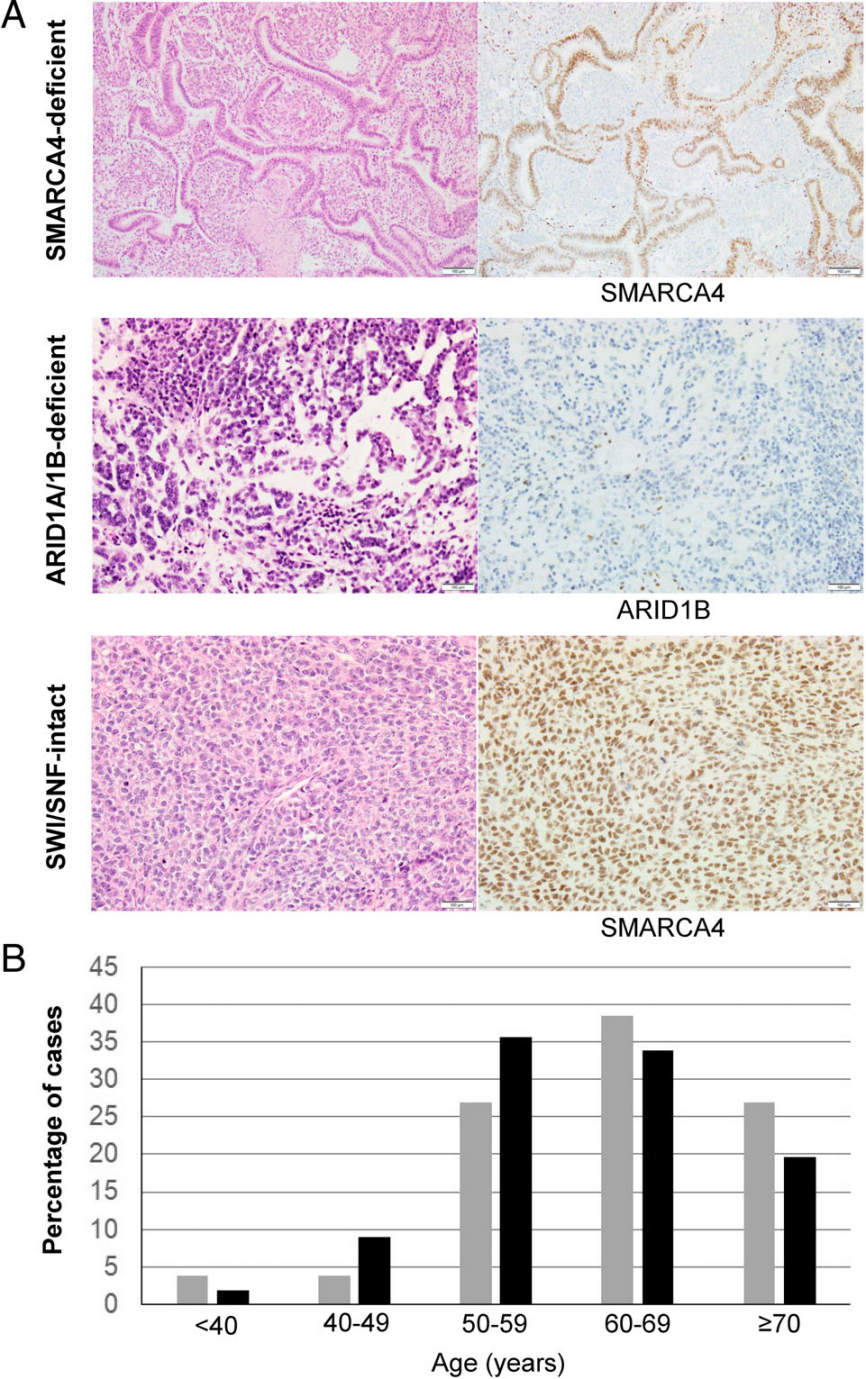
Clinicopathologic features of SWI/SNF‐deficient and SWI/SNF‐intact dedifferentiated/undifferentiated endometrial carcinomas (A). Representative histopathology and SWI/SNF protein immunostaining of SMARCA4‐deficient, ARID1A/ARID1B‐deficient and SWI/SNF‐intact tumors. (B). Age distribution of SWI/SNF‐deficient tumors (*n* = 56) (black bars) and SWI/SNF‐intact tumors (*n* = 26) (gray bars).

This study examined 56 patients with SWI/SNF‐deficient DDEC/UEC and 26 patients with SWI/SNF‐intact DDEC/UEC (Table 1). The most common presenting symptom was post‐menopausal bleeding (82%), followed by menorrhagia in 11% and abdominal pain in 5% of the patients. Of the patients with preoperative endometrial sampling, the diagnosis of DDEC/UEC was made or suggested in 41% of cases; the diagnoses in the remaining cases were variable, including grade 1 and 2 endometrioid carcinoma, high‐grade endometrial carcinoma (NOS), carcinosarcoma, high‐grade epithelioid malignancy and high‐grade endometrial stromal sarcoma. All undifferentiated tumors were composed of a proliferation of medium‐sized, monotonous, epithelioid cells growing in solid sheets with no specific pattern (Figure 1A).

**Table 1:** Clinicopathologic features of the study cohort of dedifferentiated/ undifferentiated endometrial carcinomas.

	SWI/SNF‐deficient (*n* = 56)	SWI/SNF‐intact (*n* = 26)
Age, years (range)	61 (26–85)	64 (39–93)
FIGO stage		
IA	12 (21%)	6 (23%)
IB	10 (18%)	7 (27%)
II	3 (5%)	2 (8%)
III	15 (27%)	7 (27%)
IV	16 (29%)	4 (15%)
MMR status		
Intact	18	12
Deficient	38	14
*POLE* status (exonuclease domain)		
Mutated	2 (stages I and IV)	4 (stages I, I, I and IV)
WT	54	22
SWI/SNF status		
SMARCA4‐deficient	28	0
ARID1A/1B co‐deficient	25	0
SMARCB1‐deficient	3	0
Status at last follow‐up		
DOD	38	10
AWD	0	1^*^
DOOC	0	2
NED (≥1 year)	17	11
NED (<1 year)	1	2

AWD, alive with disease; DOD, died of disease; DOOC, died of other cause; NED, no evidence of disease.

^*^ AWD at 7 months.

The average age at diagnosis for patients with SWI/SNF‐deficient tumors was 61 years (ranging from 26 to 85 years) compared to 64 years (ranging from 39 to 93 years) for SWI/SNF‐intact tumors (*p* = 0.39, *t*‐test) and the majority of patients were post‐menopausal (Figure 1B). Of the 56 SWI/SNF‐deficient DDEC/UEC, 42 were DDEC and 14 were UEC. The differentiated carcinoma components of the 42 SWI/SNF‐deficient DDEC were as follows: 32 were grade 1 endometrioid, 6 were grade 2 endometrioid, 3 were grade 3 endometrioid and 1 was serous (abnormal p53 null mutation‐type pattern in both serous and undifferentiated components). 28 tumors showed SMARCA4 loss, 25 showed co‐loss of ARID1A and ARID1B, and 3 showed SMARCB1 loss in the undifferentiated tumor. In the 14 SWI/SNF‐deficient UEC, 10 showed co‐loss of ARID1A and ARID1B, 3 showed loss of SMARCA4 (1 MMR‐deficient) and 1 showed loss of SMARCB1 (MMR‐deficient). Two SWI/SNF‐deficient DDEC/UEC harbored *POLE* exonuclease domain mutation (both *POLE* V411L hotspot) – a SMARCB1‐deficient DDEC (FIGO IA) and an ARID1A/1B‐deficient DDEC (FIGO IVB).

Of the 26 SWI/SNF‐intact DDEC/UEC, 11 were DDEC and 15 were UEC. The differentiated carcinoma components in the 11 SWI/SNF‐intact DDEC were grade 1 endometrioid in 4, grade 2 endometrioid in 4, grade 3 endometrioid in 1 and serous (abnormal p53 overexpression mutation‐type pattern in both serous and undifferentiated components) in 2 cases. Three SWI/SNF‐intact DDEC harbored *POLE* mutations (two P286R and one V411L) (all with stage I disease – pT1 and N0) and one SWI/SNF‐intact UEC harbored a *POLE* mutation (P286R) (FIGO IVB with mesenteric tumor metastasis).

With regards to MMR protein status, 37 of 56 (66%) SWI/SNF‐deficient tumors were MMR protein‐deficient (34 with loss of MLH1 and PMS2, and 3 showing isolated loss of PMS2), while 13 of 26 (50%) SWI/SNF‐intact tumors were MMR‐deficient (7 with loss of MLH1 and PMS2, 4 with isolated loss of PMS2, 1 with loss of MSH2 and MSH6, and 1 with loss of MSH6). Both SWI/SNF‐deficient tumors with *POLE* mutation were MMR‐intact. Two the 4 SWI/SNF‐intact tumors with *POLE* mutation were MMR‐deficient, with one showing isolated PMS2 loss and the other showing isolated MSH6 loss.

### Clinical outcome and response to treatment

**Figure 2:**
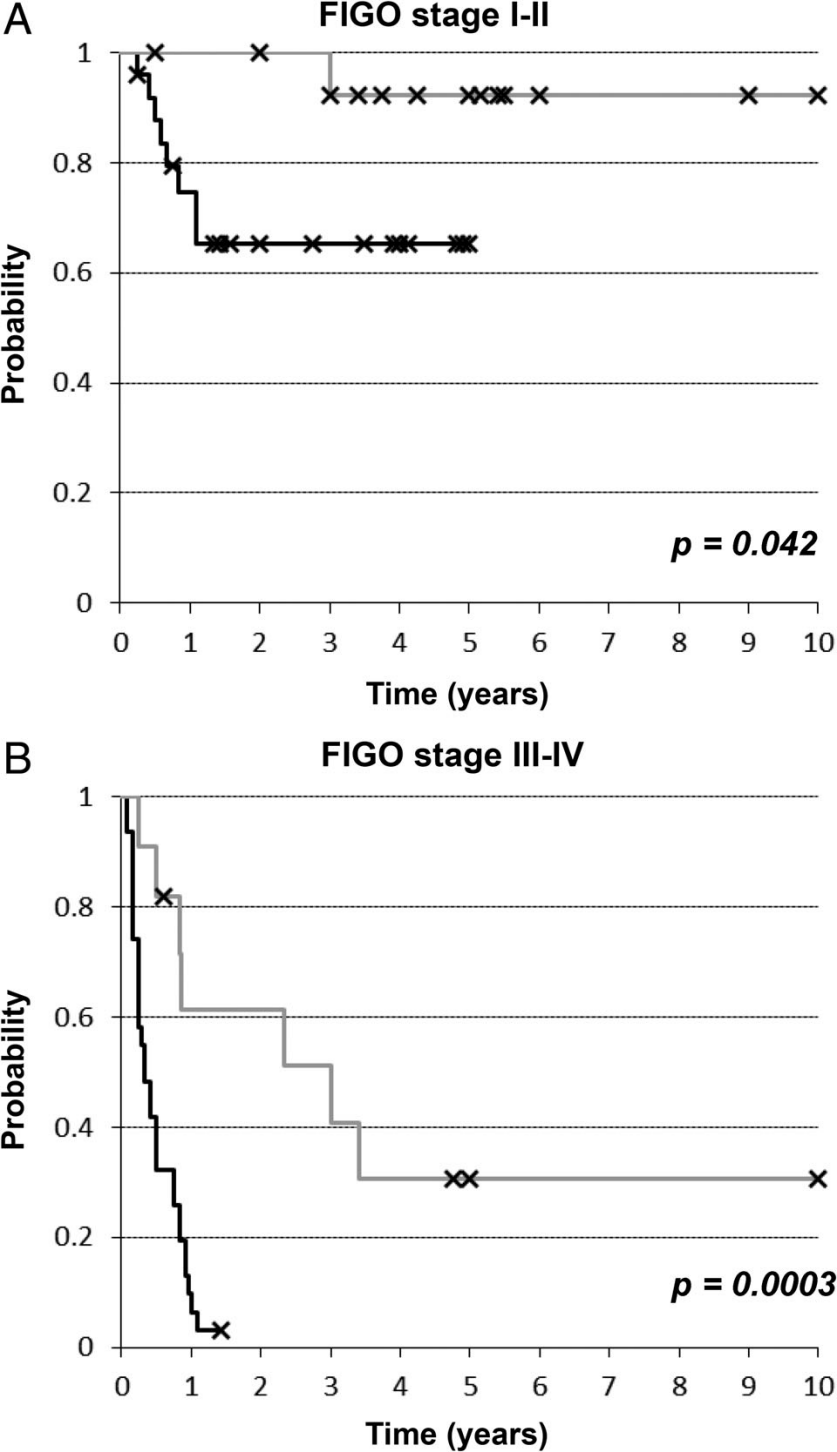
Kaplan–Meier disease‐specific survival analysis of dedifferentiated/undifferentiated endometrial carcinomas stratified by SWI/SNF protein status in (A) the early stage setting (FIGO stage I and II) and (B) the advanced stage setting (FIGO stage III and IV) (gray‐colored line: SWI/SNF‐intact tumors; black‐colored line: SWI/SNF‐deficient tumors).

In terms of tumor stage, 31 of 56 (55%) patients with SWI/SNF‐deficient DDEC/UEC presented with advanced stage (stage III or IV) disease (Table 1). 14 of the 22 stage I and 1 of 3 stage II patients had lymph node staging performed. In comparison, 11 of 26 (42%) of patients with SWI/SNF‐intact DDEC/UEC presented with advanced stage disease. The 2‐year disease specific survival (DSS) for stage I‐II disease was 65% for SWI/SNF‐deficient tumors compared to 100% for SWI/SNF‐intact tumors (Figure 2A; *p* = 0.042). All eight patients (33%) with stage I or II SWI/SNF‐deficient tumors who developed disease recurrence died from progressive disease usually within a year of initial diagnosis. In contrast, only one patient (8%) with stage I or II SWI/SNF‐intact tumors died from the disease, 3 years after initial diagnosis.

For patients with stages III and IV disease, the 2‐year disease specific survival was 3% for SWI/SNF‐deficient tumors compared to 61% for SWI/SNF‐intact tumors (Figure 2B, *p* = 0.0003), and the median survival was 4 months for SWI/SNF‐deficient tumors compared to 36 months for SWI/SNF‐intact tumors. Nearly all of the patients (30 of 31) with stage III and IV SWI/SNF‐deficient tumors died from their disease, except for one patient with stage IV ARID1A/1B‐deficient tumor that also harbored a *POLE* V411L mutation. As seen in those who experienced recurrent/progressive disease in early stage setting, all patients succumbed to their progressive disease usually within a year after initial diagnosis (with a median DSS of about 5 months). In contrast, while 7 of 11 patients with stage III and IV SWI/SNF‐intact tumors died from their disease, disease progression was slower with a median DSS of 36 months. Among SWI/SNF‐deficient DDEC/UEC, there was no significant association between MMR protein status (deficient versus proficient) and DSS in the early stage setting (2‐year DSS of 60% for MMR‐intact and 66% for MMR protein‐deficient tumors; *p* = 0.75) or in the advanced stage setting (2‐year DSS of 7% for MMR protein‐intact and 0% for MMR protein‐deficient tumors; *p* = 0.46) (data not shown).

Treatment information and accompanying clinical outcome of SWI/SNF‐deficient cases are summarized diagrammatically in supplementary material, Figure S1. Platinum‐based chemotherapy (adjuvant or neoadjuvant) was used typically in combination with taxane. Of the 22 patients with stage I disease, 3 refused adjuvant treatment; 2 of these 3 patients were alive with no evidence of disease at 33 and 42 months respectively and one experienced rapidly progressive pelvic recurrence and succumbed to her disease 13 months after initial diagnosis. Adjuvant therapy was not offered for one patient because the tumor was initially diagnosed as a FIGO grade 2 endometrioid carcinoma (pT1a without lymph node staging); 6 months after initial diagnosis she developed biopsy‐proven disseminated disease to lung with extensive mediastinal, abdominal and pelvic lymphadenopathy, and she succumbed to her disease at 8 months. Seven patients received adjuvant brachytherapy only, with one patient experiencing vaginal recurrence 6 months later; this patient refused additional therapy and succumbed to her disease 4 months after her vaginal recurrence. One patient received neoadjuvant chemotherapy (3 cycles of carboplatin and taxol) followed by interval total hysterectomy and bilateral salpingo‐oophorectomy with pelvic lymph node dissection. The uterine tumor showed no significant pathologic response to neoadjuvant chemotherapy and adjuvant chemotherapy was given. This patient was found to have recurrent/progressive disease about 2 months after her surgery (and after two cycles of adjuvant chemotherapy) with recurrent pelvic/abdominal tumor and she succumbed to her disease shortly after. Three patients received adjuvant chemotherapy and one (pT1a N0) experienced rapid disease recurrence/progression during chemotherapy with abdominal/pelvic/mediastinal lymphadenopathy and lung metastases and succumbed to her disease. The remaining seven patients with stage I disease received adjuvant chemotherapy (ranging from 3 to 6 cycles of platinum/taxane) and external beam radiation therapy. Five of these seven patients were alive with no evidence of disease over a year after initial diagnosis; the other two succumbed to recurrent disease with one patient experiencing recurrent disease during chemotherapy (radiologic evidence of paraaortic lymph node, adrenal and lung metastasis) and the other patient experiencing recurrent disease 2 months after completing her chemotherapy with progressive paraaortic/mediastinal lymphadenopathy. Of the three patients with stage II disease, one received adjuvant chemotherapy but experienced disease recurrence during therapy with abdominal lymph node and hepatic metastases; this patient succumbed to the disease 7 months after initial diagnosis. Two patients declined adjuvant treatment. One had no evidence of disease after 9 months of follow‐up and one was lost to follow‐up at 3 months with no evidence of disease.

Among the 31 patients with advanced stage disease, 9 did not receive any adjuvant therapy because of rapid disease progression (widespread metastases in 4, locoregional in 2 and details not available in 3); one refused treatment and experienced rapid disease progression, 4 received neoadjuvant platinum/taxane chemotherapy (all with stage IV disease) and none showed radiologic improvement according to RECIST 1.1 criteria with death from progressive disease within 3 months of initial presentation. 17 patients with stage III/IV disease received adjuvant chemotherapy (platinum/taxane), including 7 stage III and 3 stage IV patients who also received pelvic external beam radiation therapy, and all experienced progressive metastatic disease outside the radiation field. 12 of 17 patients experienced progressive disease during chemotherapy while 4 patients experienced disease progression within 3 months after cessation of chemotherapy. One stage IV patient with a *POLE* mutated tumor was alive with no evidence of disease after 2 years of follow‐up.

The most common sites of extrauterine tumor spread (at initial presentation or with progressive disease) included lymph nodes (abdominal/pelvic), adnexa and abdominal/pelvic soft tissue (omentum and parametrium), while metastases to lung (9 patients), adrenal gland (6 patients), brain (6 patients), bone (4 patients), and liver (3 patients) were documented in a subset of patients.

## Discussion

The existence of undifferentiated carcinoma of the endometrium has long been recognized in the pathology and clinical literature, but it was not until 2005 that Silva *et al* helped to define the diagnostic morphologic features of DDEC/UEC [5, 6, 31]. The proposed histologic definition formed the basis for the current World Health Organization (WHO) definition of DDEC/UEC. Using the histologic criteria defined, DDEC/UEC behave in a more aggressive manner than FIGO grade 3 endometrioid‐type carcinoma (a frequent histologic mimic of DDEC/UEC) [5, 31]. However, accurate distinction between solid areas of high‐grade carcinomas such as FIGO grade 3 endometrioid carcinoma, serous carcinoma, or carcinosarcoma and UEC remains a constant challenge for pathologists, both on biopsy and surgical samples. We recently identified a number of molecular alterations that appear to account for the development of UEC (dedifferentiation in the case of DDEC) [2, 11, 12]. The majority of DDEC/UEC harbor inactivating mutations involving core components of SWI/SNF complex with SMARCA4 and ARID1A/ARID1B being most commonly inactivated, resulting in absent expression of corresponding proteins in the undifferentiated tumor. These genetic insights provide the opportunity to molecularly define DDEC/UEC based on the presence of core SWI/SNF protein deficiency. In our current study cohort with centralized pathology review, we observed significant differences in clinical behavior between SWI/SNF‐deficient versus SWI/SNF‐intact DDEC/UEC. First, SWI/SNF‐deficient DDEC/UEC more frequently presents with extrauterine spread, and for patients with tumor confined to the uterus at initial clinical and surgical staging, a higher proportion develop progressive disease compared to SWI/SNF‐intact DDEC/UEC. Second, SWI/SNF‐deficient DDEC/UEC progresses more rapidly compared to SWI/SNF‐intact DDEC/UEC. If one excludes the rare *POLE*‐mutated SWI/SNF‐deficient tumors, all patients with stage III or IV SWI/SNF‐deficient DDEC/UEC succumbed to their disease within (about) a year after initial presentation. Of the patients with stage I or II SWI/SNF‐deficient DDEC/UEC (at presentation) who experienced subsequent recurrence/progression, all succumbed to the disease within (about) a year after initial presentation. In comparison, while the majority of patients with stage III or IV SWI/SNF‐intact DDEC/UEC succumbed to their disease, about half occurred more than 2 years after initial presentation, and the same slower disease progression was also observed for the small subset of stage I/II patients with progressive/recurrent SWI/SNF‐intact DDEC/UEC. This clinical trajectory for progressive SWI/SNF‐intact DDEC/UEC more closely resembles that observed for advanced stage FIGO grade 3 endometrioid carcinoma than that of SWI/SNF‐deficient DDEC/UEC [32, 33], which casts doubts whether SWI/SNF‐intact DDEC/UEC are truly undifferentiated cancer. It is likely that, despite centralized histologic review that adheres to the histologic criteria in this study, many of the cases included in the SWI/SNF‐intact group may in fact be FIGO grade 3 endometrioid‐type carcinomas. For instance, MMR‐deficient endometrioid carcinoma can sometimes display more solid growth with prominent lymphocytic infiltrates that can mimic UEC. In addition, with autolysis artifact that is seen in some hysterectomy specimens, it can be difficult to distinguish the solid growth pattern of FIGO grade 3 or even grade 2 from truly undifferentiated carcinomas based on histology alone, leading to a degree of subjectivity and variability in diagnosis. The observed difference in clinical behavior between SWI/SNF‐deficient and SWI/SNF‐intact DDEC/UEC reinforces the need for a molecular definition. We believe that a morphologically suspected UEC should be molecularly confirmed by the absence of SMARCA4, ARID1B, or SMARCB1 expression in the undifferentiated component to establish a diagnosis of SWI/SNF‐deficient DDEC/UEC which signifies a highly aggressive clinical course. It is important to note that there may be additional and novel genomic mechanisms of core SWI/SNF protein inactivation in DDEC/UEC that can be associated with the same aggressive clinical behavior as seen in SMARCA4/ARID1B/SMARCB1‐deficient DDEC/UEC. We however believe that these additional mechanism(s), if present, would only be involved in rare cases of DDEC/UEC, based on our prior studies that genetically screened 43 DDEC for mutations in all SWI/SNF complex proteins (implicated in human cancer development) which identified only genomic inactivation of *SMARCA4, ARID1B*, and *SMARCB1* as recurrent events [2, 11].

In the present cohort of SWI/SNF‐deficient DDEC/UEC, we found that all stage I/II patients with recurrent disease and all patients presenting with extrauterine tumor spread (stage III or IV disease, excluding the single stage IV case with *POLE* mutation) succumbed to their disease within a year after initial diagnosis. Long term survival is seen in the stage I/II setting only when the tumor has not recurred in the first year. Conventional platinum/taxane‐based chemotherapy appears to be completely ineffective in patients with extrauterine disease as progression during chemotherapy (in the neoadjuvant or adjuvant setting) or shortly after the completion of chemotherapy (within 3 months) was seen in all cases. The addition of pelvic external beam radiation therapy in patients with stage III disease due to lymph node involvement does not appear to prevent systemic disease spread/progression. In patients with organ‐confined disease at presentation, it is unclear whether adjuvant radiation therapy such as brachytherapy or adjuvant chemotherapy provides any survival benefit given the limited sample size and the retrospective nature of this study. However, irrespective of the use of adjuvant therapy, a subset of patients with stage I or II disease experience local or distant recurrence that is rapidly progressive. These findings clearly illustrate the ineffectiveness of platinum/taxane‐based chemotherapy in the treatment of advanced stage SWI/SNF‐deficient DDEC/UEC and underscore the need to consider other systemic therapy options. Based on the known biology of these tumors, there are two potential therapeutic approaches that warrant immediate consideration. One pertains to the MMR protein‐deficient/microsatellite‐unstable molecular context present in the majority of SWI/SNF‐deficient DDEC/UEC. Given the expanded indication for immunotherapy of MMR protein‐deficient tumors, immune check point inhibitors may be considered as first line or second line systemic treatment option for SWI/SNF‐deficient DDEC/UEC that are also MMR protein‐deficient. Moreover, three recent studies have examined the expression of PD‐L1 in DDEC/UEC and all reported PD‐L1 expression in tumor and/or stromal immune cells in about half of the cases examined, which correlated with MMR deficiency status [34, 35, 36]. Early evidence also suggests that alterations within the SWI/SNF complex could be a predictive marker of response to checkpoint inhibitors [37, 38]. However, to date there is no published preclinical or clinical evidence demonstrating that immunotherapy can improve the survival in this subset of DDEC/UEC patients. Moreover, it is unclear whether dedifferentiation alters tumor‐stromal immune cell interaction in MMR protein‐deficient endometrial carcinoma. Nonetheless, in the absence of an alternative effective systemic treatment option, immunotherapy should be urgently evaluated as treatment for this disease. Another biologically rational therapeutic approach is to exploit the presence of core SWI/SNF protein deficiency with drugs targeting chromatin remodeling/epigenetic regulation. This includes inhibitors that target polycomb complex proteins (i.e. EZH2 inhibitor). At present, there are encouraging preclinical data showing sensitivity of human tumors with core SWI/SNF protein inactivation to EZH2 inhibition [27, 28, 29]. There are also ongoing early phase clinical trials evaluating the effectiveness of EZH2 inhibition in advanced stage solid tumor and/or tumors harboring core SWI/SNF protein deficiency. Given the preclinical evidence, enrolling patients with SWI/SNF‐deficient DDEC/UEC in these trials may also be a reasonable option. However, as shown here, SWI/SNF‐deficient DDEC/UEC is frequently rapidly progressive and timely access/entry to clinical trials should be an important consideration.

It is worth noting that the recently described SMARCA4‐deficient uterine sarcoma (malignant rhabdoid tumor of the uterus) can share histologic, immunophenotypic and genetic overlap with the undifferentiated component of DDEC or UEC [25, 26]. In our present cohort of SWI/SNF‐deficient UEC, the majority (10 of 14) showed loss of ARDI1A and ARID1B, which has not been reported in rhabdoid uterine sarcoma. Among the remaining 4 SWI/SNF‐deficient UEC, 1 of 3 SMARCA4‐deficient tumors, and 1 SMARCB1‐deficient tumor were MMR‐deficient and none of the reported cases of SMARCA4‐deficient rhabdoid uterine sarcoma occur in a MMR protein‐deficient context. This leaves only 2 SWI/SNF‐deficient UEC in this cohort of SWI/SNF‐deficient DDEC/UEC with potential overlap with SMARCA4‐deficient uterine sarcoma. In the initial cohort of 5 SMARCA4‐deficient uterine sarcomas described by Kolin *et al* [25], 4 cases had advanced stage disease (stage III or IV) while staging information was not available for the fifth case (though it likely was advanced in stage as only biopsy was performed and the patient succumbed to her disease 1 month after the biopsy), and the median survival was reported as 7 months (1–43 months). Our findings here demonstrate that SWI/SNF‐deficient DDEC/UEC also behaves at least as aggressively in the advanced stage setting with median disease‐specific survival of 4 months. Aside from the demonstration of MMR protein deficiency that favors UEC, there are currently no reliable immunohistochemical markers that can be used to differentiate between SMARCA4‐deficient (and MMR protein‐intact) UEC from SMARCA4‐deficient uterine sarcoma. This includes claudin‐4 as we have shown that the undifferentiated carcinoma component of SWI/SNF‐deficient DDEC almost always lacks claudin‐4 expression [12].

Based on the results presented here, we would advocate for the use of SMARCA4, ARID1B, and SMARCB1 immunohistochemistry and *POLE* exonuclease domain mutation analysis in histologically compatible or suspected DDEC/UEC. This would enable pathologists and oncologists to identify clinically highly aggressive DDEC/UEC characterized by SWI/SNF‐deficiency and WT *POLE* status. For patients with optimally surgically staged stage I/II disease, close radiologic follow‐up with abdomen/pelvis/chest imaging is strongly recommended every 3 months for the first year, irrespective of whether adjuvant therapy is given or not. The imaging interval may be shortened for incompletely staged patients with apparent stage I/II disease. For stage I/II patients with recurrent disease and for stage III/IV patients, immunotherapy (for patients with MMR‐deficient tumors) and/or enrollment in clinical trials evaluating drugs targeting chromatin remodeling/epigenetic regulation should be urgently considered. It is important to note that our experience with SWI/SNF‐deficient DDEC/UEC harboring hotspot *POLE* exonuclease domain mutation is still very limited at present (two cases in this cohort). Further studies are needed to confirm whether *POLE* exonuclease domain mutation still imparts an excellent prognosis in SWI/SNF‐deficient DDEC/UEC, particularly in the advanced stage setting.

In summary, we observed that core SWI/SNF protein deficiency defines a clinically highly aggressive group of undifferentiated carcinomas. SWI/SNF‐deficient and *POLE* WT DDEC/UEC with extrauterine spread is universally and rapidly fatal, and conventional chemotherapy is ineffective in offering disease control. These findings underscore the need to consider defining highly clinically aggressive undifferentiated carcinoma based on core SWI/SNF‐deficiency, and to consider close follow‐up and alternative systemic treatment strategies in these cases.

## Author contributions statement

BT‐C contributed to data collection/analysis and manuscript preparation. MC contributed to study design and experimental analysis. MC, GSN, SH, RAS and CJR contributed to data collection and manuscript preparation. AL contributed to experimental analysis. LMP, MK and C‐HL contributed to study design, data analysis and manuscript preparation.

## Supporting information


**Figure S1.** Flow‐chart diagrams depicting treatment and clinical outcome FIGO stage I and II patients, and FIGO stage III and IV patients with SWI/SNF‐deficient dedifferentiated/undifferentiated endometrial carcinomas.
