# Supplementary material for: SWI/SNF‐deficiency defines highly aggressive undifferentiated endometrial carcinoma
Source: J Pathol Clin Res. 2020 Oct 30;7(2):144–53. doi: 10.1002/cjp2.188 (PMC7869930; doi:10.1002/cjp2.188)
Supplement: Supplementary file 1 — Figure S1. Flow‐chart diagrams depicting treatment and clinical outcome FIGO stage I and II patients, and FIGO stage III and IV patients with SWI/SNF‐deficient dedifferentiated/undifferentiated endometrial carcinomas. [file CJP2-7-144-s001.docx]

**SWI/SNF-deficiency defines highly aggressive undifferentiated endometrial carcinoma**

B Tessier-Cloutier *et al. J Pathol Clin Res* DOI 10.1002/cjp2.188

**Supplementary Figure S1**

**
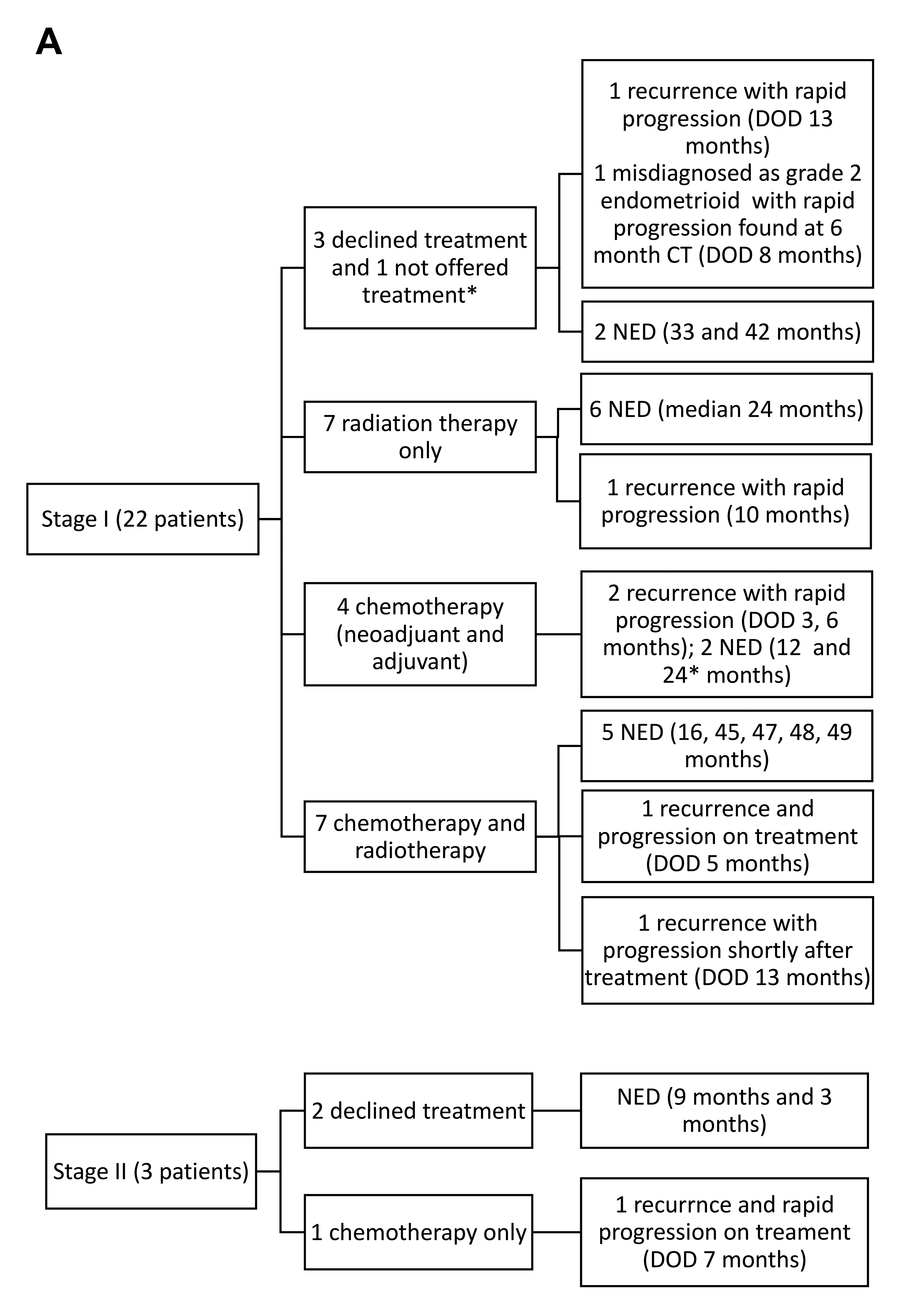
**

**
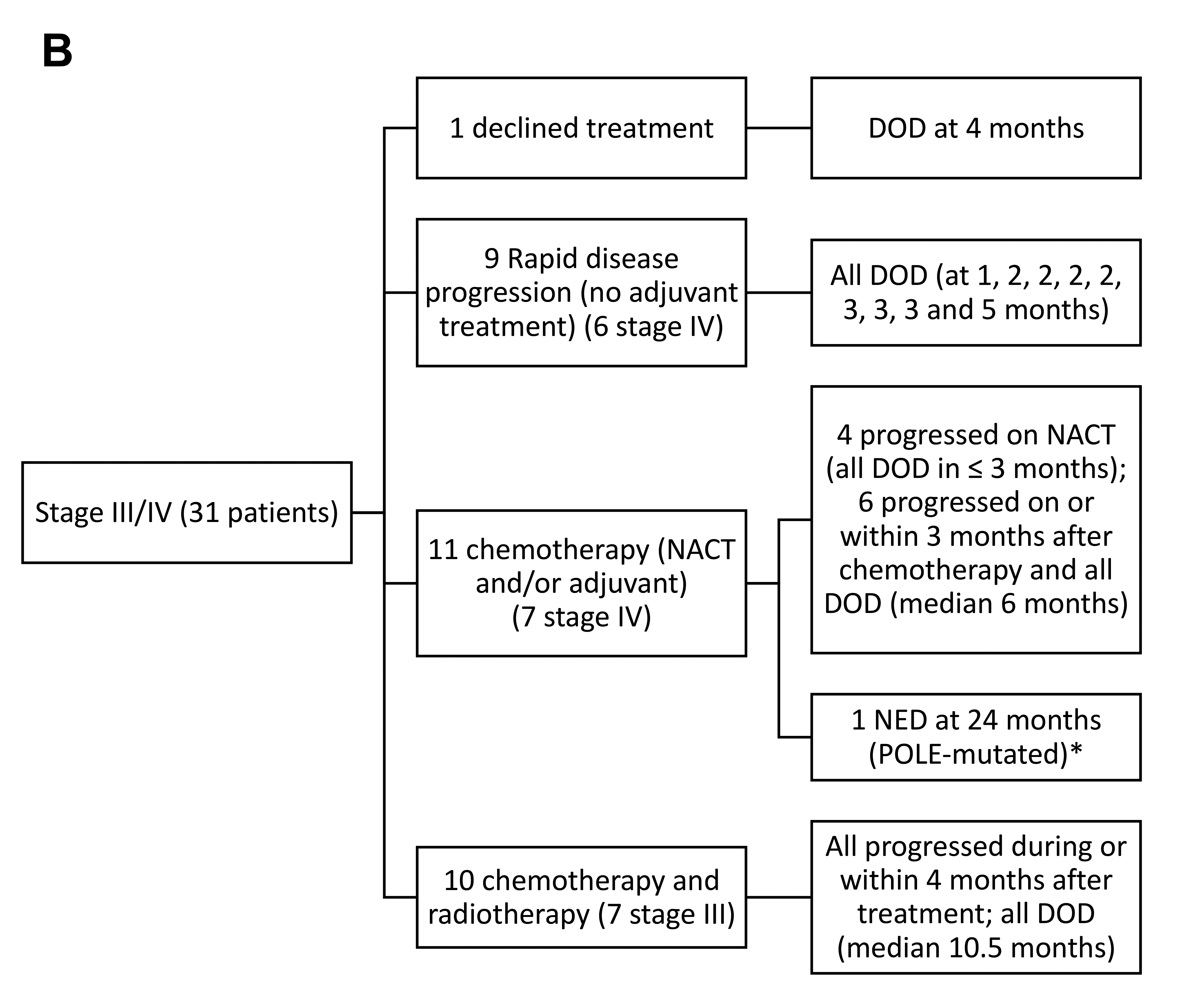
**

**Figure S1.** Flow-chart diagrams depicting treatment and clinical outcome for (A) FIGO stage I-II patients, and (B) FIGO stage III-IV patients with SWI/SNF-deficient dedifferentiated/undifferentiated endometrial carcinomas. DOD: died of disease; AWD: alive with disease; NACT: neoadjuvant chemotherapy. * *POLE*-mutated tumor.
